# Assessing the capacity of ministries of health to use research in decision-making: conceptual framework and tool

**DOI:** 10.1186/s12961-017-0227-3

**Published:** 2017-08-01

**Authors:** Daniela C. Rodríguez, Connie Hoe, Elina M. Dale, M. Hafizur Rahman, Sadika Akhter, Assad Hafeez, Wayne Irava, Preety Rajbangshi, Tamlyn Roman, Marcela Ţîrdea, Rouham Yamout, David H. Peters

**Affiliations:** 1 Department of International Health, Johns Hopkins University Bloomberg School of Public Health, Baltimore, MD United States of America; 2 World Health Organization, Geneva, Switzerland; 3 International Centre for Diarrhoeal Disease Research, Dhaka, Bangladesh; 4 Health Services Academy, Islamabad, Pakistan; 5 College of Medicine, Nursing and Health Sciences, Fiji National University, Suva, Fiji; 6 Public Health Foundation of India, Gurgaon, India; 7 Cape Town, South Africa; 8 Ministry of Health of the Republic of Moldova, Chisinau, Republic of Moldova; 9 UNICEF Lebanon, Beirut, Lebanon

**Keywords:** Government officials, Research utilisation, Decision-making, Capacity

## Abstract

**Background:**

The capacity to demand and use research is critical for governments if they are to develop policies that are informed by evidence. Existing tools designed to assess how government officials use evidence in decision-making have significant limitations for low- and middle-income countries (LMICs); they are rarely tested in LMICs and focus only on individual capacity. This paper introduces an instrument that was developed to assess Ministry of Health (MoH) capacity to demand and use research evidence for decision-making, which was tested for reliability and validity in eight LMICs (Bangladesh, Fiji, India, Lebanon, Moldova, Pakistan, South Africa, Zambia).

**Methods:**

Instrument development was based on a new conceptual framework that addresses individual, organisational and systems capacities, and items were drawn from existing instruments and a literature review. After initial item development and pre-testing to address face validity and item phrasing, the instrument was reduced to 54 items for further validation and item reduction. In-country study teams interviewed a systematic sample of 203 MoH officials. Exploratory factor analysis was used in addition to standard reliability and validity measures to further assess the items.

**Results:**

Thirty items divided between two factors representing organisational and individual capacity constructs were identified. South Africa and Zambia demonstrated the highest level of organisational capacity to use research, whereas Pakistan and Bangladesh were the lowest two. In contrast, individual capacity was highest in Pakistan, followed by South Africa, whereas Bangladesh and Lebanon were the lowest.

**Conclusion:**

The framework and related instrument represent a new opportunity for MoHs to identify ways to understand and improve capacities to incorporate research evidence in decision-making, as well as to provide a basis for tracking change.

**Electronic supplementary material:**

The online version of this article (doi:10.1186/s12961-017-0227-3) contains supplementary material, which is available to authorized users.

## Background

There is growing pressure and interest in governments using research evidence to improve their decisions, which requires both the access to evidence as well as the capacity to use it. A great deal has been learned about how to improve the communication of research findings to decision-makers, including models describing the push/pull efforts between producers and users of research [1]. However, less is known about how officials incorporate research in their decision-making, especially taking into account the broader organisational and systemic factors that influence these processes.

Individuals and organisations interact within their context regularly, and information is exchanged through multiple channels, both formally and informally [2]. It is critical to understand the individual capabilities necessary to use research and also the interactions occurring within and across organisations. Ward et al. [3] developed a conceptual framework for knowledge transfer processes that includes five domains (Knowledge/Research, Problem, Utilisation, Interventions, Contexts Barriers/Supports) that interact in a dynamic way; however, the framework does not describe the steps in each domain or their connections. Further, decision-makers are constrained by the environment that surrounds them, including complex organisational behaviour as well as procedural and regulatory parameters [4]. In order for decisions to be upheld, officials must balance the application of evidence with the larger systemic pressures they face to reach a resolution that “*maintains the stability of the system*” [4]. Recognising these forces and their role in supporting evidence use is critical because they also require specific capacities to successfully support the incorporation of research evidence in regular decision-making.

Efforts to understand how governments and decision-makers demand and use research evidence has led to several assessment tools (Box 1), though their focus has been broader than capacity for evidence use [5–8]. These existing instruments have considerable limitations, especially for low- and middle-income country countries (LMICs). Specifically, these instruments are often not written from the user’s perspective or in a language with which they relate, they focus only on the capacity of individuals, and they have been developed and tested primarily in high-income countries. The objectives of this study were to develop a conceptual framework to map the capacities of Ministries of Health (MoHs) in LMICs to demand and use research to inform policy and management decisions, and to develop and validate an instrument to assess those capacities, with the potential for comparing capacities across a sample of MoHs.

**Box 1:** Past work on assessing research demand and use in ministries of health

*Is Research Working for You? A Self-Assessment Tool and Discussion Guide for Health Services Management and Policy Organisation*
Developed by the Canadian Health Services Research Foundation (CHSRF) in 1999 to examine and facilitate discussion around the capacity of health service management and policy organisations to use research evidence in making decisions [6, 9]. Focuses on four areas of assessment: acquire, assess, adapt and apply, with questions related to these ‘four As’ and a discussion guide for the participants to discuss research utilisation in the organisation in a group setting. The tool was validated with respondents in Canada [10] and in Colombia, Argentina, Mexico and Georgia [4], and results suggest that it was useful to understand organisational capacity; however, there were questions about how to best apply the discussion portion given how useful it could be but also subject to imbalanced power dynamics among participants in hierarchical settings.
Key limitations include (1) strict focus at the organisation level, with little to no assessment of individual or systems capacities and how they influence the uptake of research evidence, and (2) despite application across country settings, government participation appears to be limited.
SUPPORT Tools
The Supporting Policy Relevant Reviews and Trials (SUPPORT) project developed a set of tools for increasing well-informed and evidence-informed decision-making targeting primarily policymakers, non-governmental organisations and civil society groups, both in low- and middle-income and high-income country settings [11]. The tools address four broad areas related to policymaking: “*1) supporting evidence-informed policymaking, 2) identifying needs for research evidence, 3) finding and assessing evidence, 4) going from research evidence to decisions*” [11]. As part of the SUPPORT Tools, a specific tool for organisational capacity to support the use of research evidence to inform decisions was also developed [8]. The tool consists of seven sections that assess:
• Organisational culture and values to support the use of research evidence to inform decisions
• Setting priorities for obtaining research evidence
• Obtaining research evidence
• Assessing the quality and applicability of research evidence and interpreting the results
• Using research evidence to inform recommendations and decisions
• Monitoring and evaluating policies and programmes
• Supporting continuing professional development
It is not clear whether the SUPPORT Tools have been formally tested for reliability and validity, or whether there is a standard method for involving particular types of participants to obtain more standard measures and benchmarks. For the specific tool on organisational capacity, the focus is on the organisation with no specific assessment of individual or systems capacities.
*Data Demand and Information Use (DDIU) in the Health Sector*
Developed by MEASURE Evaluation focusing on generating and collecting health information data with a view towards informing policymaking [7]. The ‘Checklist for DDIU Assessment’ is not a fixed instrument but provides guidance on assessing technical, organisational and behavioural/individual constraints that can affect the demand and supply of data. The approach is not prescriptive and specific, but asks broader questions about potential barriers to collecting, sharing and using data. While DDIU takes on individual, organisational and systems capacities, its focus is on generation and use of data rather than research evidence.
*Other Instrument*
Boyko et al. [5] developed an instrument based on the theory of planned behaviour to evaluate the intention of policymakers to use research. The instrument was tested with Canadian policymakers and stakeholders who had participated in deliberative dialogues about relevant topics, and was found to be reliable. However, due to the small sample size, the instrument’s validity could not be assessed. This instrument is focused almost exclusively at the individual level, with assessments of individual attitudes and expectations towards use research. Additionally, it is not clear whether this instrument would be applicable in other settings, especially in low- and middle-income countries.

It is very rare for a specific example of research evidence to result directly in a policy decision. Thus, our approach to ‘demand and use research evidence’ is meant to be broad enough to account for the many ways that individuals and organisations use information – not only instrumental or rationalistic use, but enlightenment and symbolic use as well [12, 13]. In an effort to acknowledge the larger organisational and systemic pressures that influence how officials demand and use research evidence, we adopted the definition of capacity used by the United Kingdom’s Department for International Development (DFID), which takes into account capacity at the level of individuals, organisations and systems [14]. In the context of this study, we are using ‘systems’ to imply the ability to engage across organisations and in broader society with the aim of improving evidence use. Overall, capacity is influenced by the interdependence and interaction of the three levels within the surrounding environment, making it both relational and political [15, 16].

This paper describes the development of the conceptual framework that focuses on the internal processes at MoHs and the interactions across the three levels of capacity, and we present an instrument designed to assess MoH capacity to demand and use research evidence for decision-making, including the results from testing in multiple LMICs.

### Conceptual framework

The foundation for this new instrument is a conceptual framework based on an extensive literature review conducted between October and December 2011 to capture research articles and reports addressing capacity to use research. At the outset, key concepts including capacity, research evidence, utilisation/research utilisation and decision-making were first defined by the research team from existing literature. Two complementary approaches were used to identify potential publications, namely (1) a search of two databases of published literature (PubMed and SCOPUS), and (2) hand searching of the table of contents of relevant journals and databases for grey literature on economic development between 2001 and 2011 (Table 1).

**Table 1:** Journals and databases that were hand searched

Peer-reviewed journals	Economic development databases
Health Policy and Planning	3ie
Health Research Policy and Systems	EuropeAid
Implementation Science	GIZ
Milbank Quarterly	OECD
Public Administration Review	UKaid
Social Science and Medicine	USAID
WHO Bulletin	

Initial results yielded 1015 articles/reports (926 from databases, 89 hand searched), with 1007 remaining after de-duplication. Only literature that focused on (1) medium to large health organisations (i.e. more than 20 employees), (2) addressed evidence collected systematically (with clear and reproducible methodologies), and (3) either addressed research utilisation or capacity concepts, were included for review; this yielded 116 articles for abstraction (Additional file 1).

The articles were abstracted into a database to include identifying information (e.g. authors, title, publication, language, funder, etc.); research question, methods and results; constructs or theories guiding the research; and whether reliability or validity testing was conducted. Given the project goals of developing a conceptual framework and assessment tool, any articles with a conceptual framework or instrument/tool were flagged during abstraction. Articles were ranked by the abstractor for their usefulness and applicability to the project goals on a 1–3 scale. Articles were abstracted by four different abstractors who had pretested the abstraction form for consistency, and rankings were reviewed as a team.

Fifteen articles with the highest ranking plus 14 others with frameworks and/or instruments were reviewed in depth for development of the conceptual framework (Additional file 2). Each publication was further reviewed to assess use of evidence at individual, organisation or systems level, capacity assessment or needs, and use of tool or framework. Most of the 29 publications focused on evidence-based decision-making and factors that influence the use of evidence, including capacity needs (e.g. [17–24]), while others focused specifically on capacity-building or assessments (e.g. [5, 8, 10, 25–27]).

**Fig. 1:**
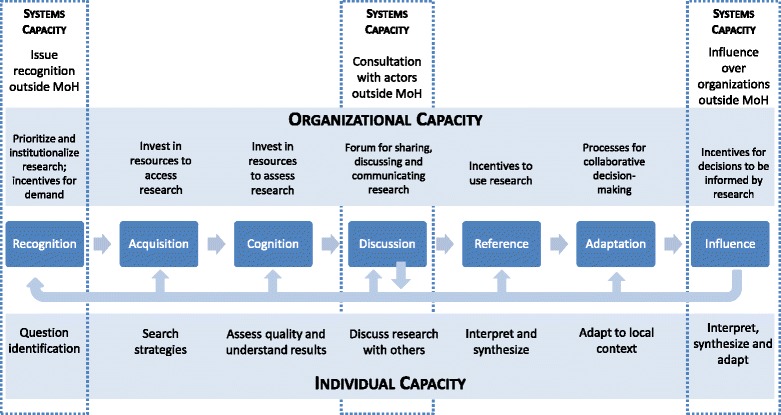
Conceptual framework for the Ministry of Health’s capacity to demand and use of research evidence

Two articles were especially useful because they addressed in detail aspects of capacity relevant to evidence use. Landry et al. [20] studied the use of university research by government agencies in Canada based on ‘Steps of Knowledge Utilisation’, namely reception, cognition, discussion, reference, effort/adaptation and influence. A key concept in this approach is that the steps are cumulative and an individual cannot reach a late step in utilisation without having gone through all of the earlier steps. In this study, government officials in several domains were assessed on their experiences using university research in their work [20]. This approach is very focused on the individual’s experience, with little to no organisational or systems assessment. However, this is one of the few frameworks that focuses on the various steps that intervene between acquiring evidence and effectively integrating it into day-to-day work activities. Potter and Brough’s work [28] on the pyramids of capacity and capacity-building is focused on building a hierarchy of needs to inform systematic capacity-building. Although the focus is more specifically on enhancing capacity in development programmes, we used this work to inform the organisational and systems’ levels of our conceptual framework and instrument. In particular, we drew on the work on structural capacity (e.g. inter-sectoral interactions) and role capacity (e.g. authority to making decisions) [28]. Our conceptual framework brings together all of these elements together into one interactive space (Fig. 1).

The framework defines MoH capacity to demand and use research evidence to inform policy and management decisions operating on three levels, namely individual, organisational and systems levels.

1. Individual competencies: people in MoHs need the skills to identify and interpret research evidence.
2. Organisational capabilities: MoHs need structures, practices, and resources that support the demand and use research evidence in its decisions.
3. Systems interactions: MoHs need the leadership and processes through which the MoH addresses the broader policy environment, and influences society and organisations beyond the MoH.

The framework operationalises these areas of capacity by disaggregating the process of demanding and using research evidence into seven distinct steps, namely recognition, acquisition, cognition, discussion, reference, adaptation and influence (Fig. 1). Based on our review, we found that it was important to break down the ‘cognition’ step from Landry et al. [20] into two separate steps that acknowledged the distinct needs related to acquiring research evidence.

Under this framework, recognition and acquisition represent the demand for research evidence, whereas the subsequent steps focus on the use. Individual and organisational capacities have a role in every step, while systems capacities play a role only at recognition, discussion and influence. Although these steps are represented in a linear fashion in the framework, we have attempted to highlight the interaction between steps with arrows illustrating iterative feedback loops.

The individual competencies needed for each step are:

1. Recognition: has the motivation to use research and can identify questions that can be answered by research evidence;
2. Acquisition: knows where and how to search for research evidence;
3. Cognition: can assess quality of research and understand results;
4. Discussion: discusses research evidence with colleagues, researchers and others;
5. Reference: ability to interpret and synthesise research evidence;
6. Adaptation: ability to adapt results to local context or current question; and
7. Influence: sufficient latitude within their role to use research evidence to influence decisions.

In terms of the organisational capabilities, the categories described here are broader because they have different manifestations across each step of the framework.

- Value:
  - Prioritise research utilisation
  - Institutionalised in organisational culture
- Expectation:
  - Structure in place to (dis)incentivise research utilisation, including monitoring and evaluation to ensure that research is incorporated in decisions
  - Peer expectations and influence
- Infrastructure:
  - Investment to ensure research utilisation takes place, including funding, staff time, technical resources (hardware, software, IT support), facilities
  - Access to experts when necessary
  - Skills development/investment through trainings and mentoring
- Process:
  - Clear processes for research evidence to be introduced into decision-making processes, be shared and discussed, including discussion fora (ongoing and for specific issues) and staff participation
  - Communication processes around research utilisation

Finally, systems interactions are described in this framework as they relate to interactions beyond the MoH (as distinct from the systems to use research within a MoH that are included at the organisational level in this framework).

- Issue recognition: structures and processes whereby actors outside of the MoH draw attention to existing or emergent issues
- Consultation: formal and informal mechanisms for consultation between MoH actors and outside actors around agenda items that are driven by research evidence
- Outside influence: processes whereby MoH attempts to influence outside audiences regarding their behaviour, priorities and decisions, specifically those driven by research evidence

## Methods

### Instrument development

In order to capture the constructs of the conceptual framework completely, items were developed by (1) adapting items from existing instruments found in the literature review, (2) adapting items from earlier work by members of the research team, and (3) creating new items. The instrument contained an initial set of 160 items. The research team members each completed a copy of the instrument, which was followed by a debriefing session where the team discussed as a group whether the questions were understood as intended. Revisions to the instrument were made, and the number of items was reduced to 90 to ensure that each concept identified in the framework was represented and to reduce item duplication.

A small pre-test of the instrument was then conducted. Five individuals with extensive experience working with or within MoHs in LMICs were recruited to complete the instrument. Each individual was asked to answer questions in the instrument after which a debriefing session was held with each individual. Debriefing sessions explored whether the experts understood the questions as intended, and the extent to which the items could be generalised to all possible items of that domain. Respondents were also asked to rate questionnaire items based on whether they believed the item was a valid measure of capacity to demand and use research.

Further revisions were made based on the pre-test, including dropping items that did not reflect the constructs well, resulting in 54 items plus five new demographic questions. The items include Likert scales, multiple-choice questions and a limited number of open-ended questions. The open-ended questions were expected to provide a basis for interpretation of the other aspects of the questionnaire, in particular during testing. In total, 11 items were drawn directly from existing instruments, 15 were adapted from existing instruments, and 28 were new but related to the conceptual framework.

### Instrument components

The draft instrument was divided into three sections that correspond to the three levels of capacity included in the conceptual framework, namely individual, organisational and systems levels. However, there was some cross-over between capacity types in each section in order to ease the flow of administration.

Section one contained 35 items focused on the MoH’s organisational capacity to use or support the use of research evidence, including the value, expectations and attitudes of the MoHs about using research evidence in decision-making. In addition, there were questions about the processes for using research evidence in the MoH, and about how the MoH reaches out to others on issues that are influenced by research evidence.

The second section included seven systems items exploring the relationship and activities between the MoH and stakeholders outside the Ministry around using research evidence to influence decisions. Stakeholders were defined as a broad range of individuals and organisations outside of the MoH who are involved in, affected by or engage in decisions made by the Ministry, including other government agencies, non-governmental and civil society organisations, and professional organisations.

The last section (individual capacity) contained 12 items related to how individuals at the MoH integrate research evidence in their work activities, including attitudes and expectations about using research evidence. There were also questions about which sources of research individuals refer to, any skills and training they have received to use research evidence, and resources available at the unit level to support the use of research.

### Study design

The study was conducted in collaboration with the WHO Alliance for Health Policy and Systems Research, which supported the identification of study countries and contracted local teams for data collection. Given the nature of the study, it was necessary to apply the instrument in English across all countries and respondents in order to validate the instrument.

#### Sampling framework

The original intent was to include 12 countries with equal distribution across WHO regions. There were significant challenges in identifying countries where MoH officials could complete the instrument in written English, and where there was a country team with the ability to collect data in their MoH. The final study sample is shown in Table 2.

**Table 2:** Study countries

Countries	WHO Region	Economic status^a^	Population (Thousands)
Fiji	Western Pacific	Lower middle income	868
Moldova	Europe	Lower middle income	3559
South Africa	Africa	Upper middle income	50,856
Zambia	Africa	Lower middle income	13,747
Lebanon	Eastern Mediterranean	Upper middle income	4259
Bangladesh	South-East Asia	Low income	150,493
India	South-East Asia	Lower middle income	1,241,491
Pakistan	Eastern Mediterranean	Lower middle income	176,745

^a^Source: World Bank World Development Indicators. All data 2011

Recognising the variability of the MoHs organisational structure, a general, standard rubric was developed to identify potential respondents (Table 3). In order to be applied across contexts, the rubric was used to identify MoH staff in units that have a direct decision-making mandate to support health system components and health topics. Due to the nature of the instrument, the rubric focused on:

**Table 3:** Sample rubric for study respondents

MoH Unit	Type of respondents^a^	Number per unit
Ministerial level	Minister, Vice/Deputy Minister, Administrative head (e.g. Principal Secretary) and Head of Health Service (e.g. Director General) or their equivalents	3–5 persons per MoH, depending on organisation structure
Policy and planning unit (or equivalent)	Head and Deputy Head	2 respondents
Research unit (if one exists)	Head and Deputy Head	2 respondents
Monitoring and Evaluation unit	Head and Deputy Head	2 respondents
Public health programmes	Head and Deputy Head	2 respondents
Specific health programmes, if multiple programmes (e.g. maternal/child health, nutrition, HIV)^b^	Head of programme (random selection of 5 respondents from heads of such programmes)	5 respondents
Hospital services (or health services)	Head and Deputy Head	2 respondents
International relations	Head of unit	1 respondent
Regional/district units	Head of unit (random selection of 10 respondents from heads of such units)	10 respondents

^a^For those units that have two Deputy Heads, ask each to participate. If there are more than two Deputy Heads, randomly sample two of them

^b^In the specific health programmes, identify at least four or five eligible divisions

- MoH officials at the central level departments and divisions (not regional and sub-regional levels);
- MoH officials who have responsibilities that are directly health-related;
- MoH officials operating under the political leadership of the MoH.

Further, the rubric was used to identify individuals who inform decision-making processes through their work; or who commission, conduct or use research; or have the authority to mandate others to do any of these things. The rubric was used to identify 25–30 respondents per study country, with an additional 5–7 back-up respondents.

#### Data collection

Data collection teams were engaged in each study country. Teams ranged from one to four persons, and represented academic/research institutions, independent consultants and government entities, all with multi-year experience engaging with MoHs.

In order to ensure consistent application of the instrument, a standardised protocol was developed and module-based training was conducted with the country teams, which included both self-guided modules as well as interactive sessions via webinar covering relevant topics, such as familiarisation with the study concepts and materials, practice session reviewing and applying the tool, and an interactive session for discussion. Country teams were grouped into three training cycles based on availability and time zone. Each training cycle was designed to last 4 weeks, though in practice it took up to 8 weeks due to scheduling conflicts.

Data were collected in-person by country teams through individual interviews with respondents. Upon completion of the instrument, country researchers entered the data on a web-based version of the instrument. In order to help assess reliability of the instrument, interviewers also asked respondents a series of reflection and exit interview questions. Data were collected between September 2013 and June 2014.

#### Data analysis

Data were analyzed to assess validity and reliability of the instrument itself and the administration methods. Analyses were conducted in STATA and MPLUS.

The original instrument included three types of response options, namely multiple choice, binary options and Likert scale agreement responses. In order to standardise the items for analysis, all Likert scale items were revised so that analysis could be made on binary outcomes. In other words, ‘Strongly Agree’ and ‘Agree’ were combined, and ‘Disagree’ and ‘Strongly Disagree’ were combined to create a binary response for each item. These adjustments resulted in the original 54 items becoming 79 items during analysis.

#### Validity

In broadest terms, validity of a questionnaire is the extent to which it measures what it claims to be measuring – in this case, the true definition of the capacity of MoHs to use research. Although experts were used to assess the ‘face validity’ of the instrument items, the strongest assessments of the validity of an instrument depend on comparison to a gold standard. Given the absence of such a standard for this topic, exploratory factor analysis (EFA) was used instead to assess how different items align into common conceptual constructs that describe different elements of capacity to use research.

EFA using the oblique GEOMIN rotation was conducted on the 79 items of the instrument. Given that responses were measured on ordinal scale, the model was fit to a polychoric correlation matrix using the method of robust weighted least squares. Standard error computations used a sandwich estimator in order to account for non-independence of observations.

To determine the appropriate number of factors to retain several criteria were used. First, Kaiser-Guttman criterion was used (eigenvalues >1). This was coupled with evaluation of the proportion of total variance that was explained by given factors. Parallel analysis was not applicable as the data was measured on a binary scale.

Next, model fit statistics, factor loadings, communalities and factor structure were examined. Given the sensitivity of the χ^2^ test to sample size, the comparative fit index, Tucker–Lewis index, the root mean square error of approximation and the standardised root mean residual were used to evaluate the global fit of the model. The use of several indices to judge the model fit is recommended as each of them has its strengths and weaknesses.

Overall, decisions regarding the number items that need to be dropped were taken in conjunction with the reliability test results.

#### Reliability

A series of steps were taken to assess whether the instrument was internally consistent or reliable.

1. Interpretation verification. Respondents were asked open-ended questions at the end of each section to understand their interpretation of the tool, including relevance of the items and response options to their experience, and identification of items with confusing or inconsistent meanings. Further, a high proportion of missing values (e.g. > 10% missing) also indicates problems with understanding of the items or their relevance.
2. Test-retest reliability was calculated to assess reliability of the responses over time. A sub-sample of each country’s respondents (*n* = 4) was randomly selected for retest. The period for retest ranged between 1 day and 6 months, with most retests occurring within 1 month.
3. Internal consistency. Item-total correlation and Cronbach’s alpha based on polychoric correlation matrix were used to estimate the internal consistency of the scale. Items with item-total correlation below 0.2 would be removed, as defined in standard practice [29]. Further, we aimed to achieve a Cronbach’s alpha of 0.70–0.90, with 0.7 often considered the minimum. It is often noted, however, that alpha coefficient is sensitive to the number of items in a questionnaire [30].

## Results

### Study sample

Across the eight study countries, there were 203 responses. Six out of eight countries were able to conduct secondary follow-up administrations to a subsample of respondents (retest) for a total of 24 (Table 4).

**Table 4:** Number of test and retest respondents by country

Countries	Initial respondents	Retest
Fiji	24	4
Moldova	25	4
South Africa	20	0
Zambia	26	0
Lebanon	26	4
Bangladesh	24	4
India	30	4
Pakistan	28	4
Total	203	24

### Preliminary analysis

1. Items were reviewed for missingness first – proportion of missing values by item and across all items was very low (below 5%). Next, frequency distribution of responses was assessed. Items with high endorsement rate were examined further, and four items were dropped.
2. Based on qualitative review of the responses to the reflection and exit interview questions, it was determined that four items asking about resources were difficult for respondents to interpret accurately and the response options were felt to be inadequate. Also, the eight questions around ‘systems’ capacity were found to be well-understood but lack adequate response options to capture the in-country experience. Finally, one item about training to use research evidence was felt not to capture respondents’ experiences with formal training.
3. Test-retest correlations suggested that 23 items were candidates for removal. Nine items had *P* values greater than 0.05, 11 items had correlations less than 0.8, and three items could not be computed (Additional file 3). Of these, 22 were eventually dropped.

### EFA results

**Fig. 2:**
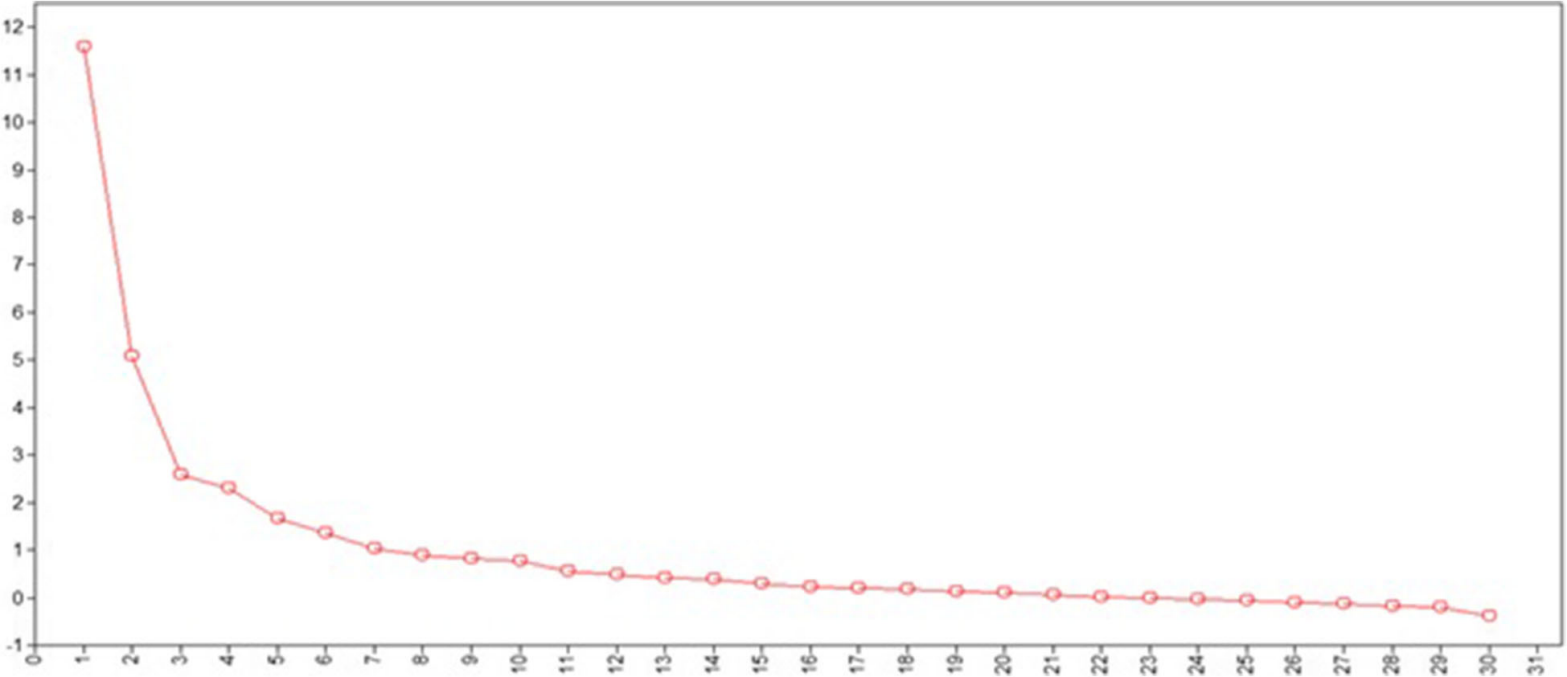
Scree plot

The scree plot (Fig. 2) suggested a three-factor model (64% variance explained). Upon review, two out of the three factors had a cohesive conceptual explanatory value and the remaining factor was a collection of items that could not be cohesively explained. The decision was made to limit the model to two factors explaining 56% of the variance. The fit indices for the two-factor model provided evidence of a good fit: the comparative fit index was 0.922, Tucker–Lewis index was 0.910, and the root mean square error of approximation was 0.045 (90% CI 0.022–0.045).

Items with loadings below 0.4 were dropped. Items with cross-loadings between factors with a difference of less than 0.2 were dropped. Items with cross-loadings between factors with a difference greater than 0.2 were placed in the factor with the highest loading.

### Validity and reliability

1. Internal consistency: Item-total correlations were calculated, all of the items had a correlation above 0.2, which is sometimes recommended as a criterion for item exclusion [31].
2. Cronbach’s alpha coefficient was estimated to assess internal consistency for the two factors: 0.89 for Factor 1 and 0.83 for Factor 2.

As a result of the above analyses, the instrument was refined to 30 items divided between two factors (Table 5).

**Table 5:** Final list of items by factor

Original item no	Item text	F1^a^	F2^a^
1	Using research evidence is a priority in the MoH	0.883	
2	Leadership in the MoH supports evidence-informed data	0.951	
3	Decision-makers in the MoH give consideration to any recommendations based on research evidence	0.694	
4	There is a transparent process for how research evidence is used in decisions at the MoH	0.678	
8	Has the MoH conducted any activities to promote the use of research evidence in the last year?	0.476	
10	The MoH has a process to check regularly whether I use research evidence in my work	0.607	
14a	Our staff has enough time to evaluate research evidence		0.736
14c	Our staff has enough resources to evaluate research evidence		0.684
15a	Our staff has enough time to compare what the MoH does to what the research evidence says		0.924
15c	Our staff has enough resources to compare what the MoH does to what the research evidence says		0.791
16a	Our staff has enough time to link research evidence to key issues facing decision-makers		0.944
16c	Our staff has enough resources to link research evidence to key issues facing decision-makers		0.972
17a	Our staff has enough time to provide recommendations based on research evidence to decision-makers		0.860
17c	Our staff has enough resources to provide recommendations based on research evidence to decision-makers		0.961
18a	Who does this for the MoH?… – Search for and retrieve research evidence – MoH staff	0.782	
19a	Who does this for the MoH?… – Interpret research evidence – MoH staff	0.925	
20a	Who does this for the MoH?… – Synthesise into one document all the relevant research evidence, information and analyses for a specific issue – MoH staff	0.829	
21a	Who does this for the MoH?… – Compare what the MoH does to what the research evidence says – MoH staff	0.831	
22a	Who does this for the MoH?… – Link research evidence to key issues facing decision-makers – MoH staff	0.823	
23a	Who does this for the MoH?… –Provide recommendations based on research evidence to decision-makers – MoH staff	0.815	
26	The MoH gets involved with researchers as partners in decision-making	0.627	
31	The MoH has a good process to advocate its priorities based on research evidence to the public, such as to promote behaviour change	0.749	
32	The MoH has a good process to advocate its priorities based on research evidence to health workers, such as to promote changes in clinical practice	0.844	
33	The MoH has a good process to advocate its priorities based on research evidence to other ministries, such as to justify the costs of health interventions	0.600	
34	The MoH has a good process to advocate its priorities based on research evidence to professional organisations, such as to promote new roles for different health workers	0.661	
35	The current policy environment is supportive of the MoH using research evidence for its decisions	0.607	
36	The current government is supportive of the MoH using research evidence for its decisions	0.557	
37	Stakeholders outside the MoH actively engage the MoH to contribute research evidence to inform decisions	0.586	
50	Our unit has regular access to a computer for acquiring and analysing research evidence		0.631
51	Our unit has regular access to the Internet at work for accessing research evidence online		0.706

^a^Significant at < 0.05

*MoH* Ministry of Health

The two factors represent two of the original areas of capacity (organisational and individual) that were identified within the conceptual framework that formed the basis for the instrument. Only three of the systems capacity items were retained and these fell within the first factor on organisational capabilities, so the capacity areas have been defined as follows:

- Organisational capabilities (Factor 1): processes and practices that reflect MoH commitment to using research evidence in its decisions and for influencing others outside the MoH, including allocation of staff to take specific steps in its use.
- Individual capacities (Factor 2): access to sufficient resources necessary for MoH staff to assess evidence and provide recommendations.

The final items were mapped onto the seven steps in demand and use of research evidence described in the conceptual framework (Table 6). All of the steps in the conceptual framework are represented among the final items suggesting that content validity has also been achieved.

**Table 6:** Distribution of final items between seven steps from conceptual framework to demand and use research evidence

Conceptual framework step	Organisational capabilities	Individual capacity	Total
	Example	Example	
Recognition	Using research evidence is a priority for the MoH	–	6 (6 ORG)
Acquisition	MoH staff search for and retrieve research evidence	Our unit has regular access to the Internet at work for accessing research evidence online	2 (1 ORG+1 IND)
Cognition	–	Our staff has enough time to evaluate research evidence	3 (3 IND)
Discussion	The MoH gets involved with researchers as partners in decision-making	–	1 (1 ORG)
Reference	MoH staff synthesise all the relevant research evidence, information and analyses for a specific issue	Our staff has enough resources to compare what the MoH does to what the research evidence says	5 (2 ORG+3 IND)
Adaptation	There is a transparent process for how research evidence is used in decisions at the MoH	–	2 (2 ORG)
Influence	The MoH has a good process to advocate its priorities based on research evidence to the public, health workers, etc.	Our staff has enough resources to provide recommendations based on research evidence to decision-makers	11 (7 ORG+4 IND)

*MoH* Ministry of Health, *ORG* organisational, *IND* individual

For ease of use, a country completing the instrument would be able to identify a high performance or low performance based on the score received. Two approaches were tested for ranking purposes – mean score and standardised z-score. For the mean score, a simple scoring protocol of 0–1 for each item was used, generating a mean score per country. For the standardised z-score several steps were required to standardise around the entire sample – (1) estimating the scale for respondents based on a simple summation across the 30 items (range 0–30), (2) estimating z-scores for each individual based on the grand mean, and (3) using the simple mean of individual scores to get a country score.

Based on this study, South Africa was the highest performer in capacity to demand and use research evidence and Bangladesh was the lowest consistently across ranking approaches (Table 7). However, when the factors were considered independently, the results varied considerably for Factor 2 (Individual Capacities), with Pakistan moving from the last position to the first (Table 8).

**Table 7:** Ranking of study countries

Country	Mean score	Standardised Z-score	Total respondents
South Africa	24.2	0.72	20
Zambia	21.8	0.35	26
India	20.8	0.19	30
Fiji	20.4	0.12	25
Moldova	20.4	0.12	20
Lebanon	17.6	–0.32	27
Pakistan	16.5	–0.48	28
Bangladesh	16.3	–0.53	24

**Table 8:** Ranking of study countries by factor

Factor 1		Factor 2	
Country	Mean	Country	Mean
South Africa	18.4	Pakistan	6.0
Zambia	16.4	South Africa	5.9
Fiji	16.1	Zambia	5.4
India	16.0	Moldova	5.2
Moldova	15.2	India	4.9
Lebanon	13.3	Fiji	4.4
Bangladesh	12.1	Lebanon	4.3
Pakistan	10.5	Bangladesh	4.1

## Discussion

This article presents a new, comprehensive conceptual framework to understand and assess the individual, organisational and systems capacities needed for MoHs to demand and use research evidence, and presents results from testing a tool to assess capacity based on the conceptual framework. The conceptual framework breaks down the overarching construct of ‘research evidence use’ into seven discrete steps, making it easier to assess evidence use, but more importantly identifying areas for building skills and capabilities. Rather than seeing these as strictly stepwise in function, the interactions between steps seem to be more iterative, and strengthen the entire process.

The results suggest that organisation and individual capacities remained important among the concepts assessed by the tool but the items around systems capacity appeared to have less prominence. The systems items were also meant to address larger contextual issues but they did not explain much of the variation in the EFA, and may have therefore not played as important a role in determining use of evidence compared to organisational and individual factors. Study countries represent a wide spectrum of political and cultural systems and thus these results suggest that larger decision-making dynamics perhaps do not influence an individual Ministry as expected. However, we did not pose specific situations where inter-organisational issues may have been more important; hence, in the proposed tool, we have retained aspects of the systems capacities through open-ended discussion questions at the end of the tool to ensure that issues around external stakeholder engagement are addressed (Additional file 4). Further research could use this tool to examine the extent to which broader contextual factors affect evidence use over time across different regime types.

There is limited evidence across LMIC settings of how to best improve evidence use through capacity-building. A recent multi-country evaluation of capacity strengthening interventions also took the approach of assessing capacity on three levels, namely individual, organisational and institutional levels [32]. Country teams in Bangladesh, Gambia, India and Nigeria conducted activities to improve policymakers’ capacity to use evidence. The evaluation found that most activities were aimed at building individual capacity and were quite successful, while other, more limited activities aimed at organisational and institutional capacity were more challenging. Specifically, results from India suggested that building the capacity of interested outsiders (e.g. civil society) built overall systems capacity by demanding evidence-informed decisions. These findings support a multi-level conceptualisation of capacity to use evidence, which may require multi-level interventions to provide overall lasting improvements where organisational and institutional capacity may be the most difficult to build. Future research should also explore the interaction between individual, organisation and systems capacity levels, and how they may enhance or inhibit each other.

The tool presented here would be most useful if Ministries self-initiate the assessment as a way to generate productive discussions around capacity for research utilisation, identifying areas for improvement and taking necessary steps to build capacity. Study results indicate that the overall tool was consistent in ranking capacity to demand and use research evidence among study countries; however, rankings did vary when considering each factor independently. In the case of Pakistan, this meant that despite a high individual score, it ranked relatively low due to its low organisational score. The combined score gave greater prominence to the organisational capacities; we suspect that strong individual skills would not have much impact within an organisational context that does not support evidence-informed decision-making, and study results support this. However, separate scores for organisational and individual capacities are useful to examine the particular strengths and weaknesses of a country.

These results have implications for how to structure efforts to improve a Ministry’s capacity. Targeting individual capacity through training, for example, needs to be coupled with addressing leadership needs and processes aimed at improving organisational capabilities but the mix of approaches will and should vary by country. The relationship between the instrument items and the conceptual framework steps forms the basis for generating a greater understanding of a MoH’s capacity in these areas and makes it easier for stakeholders to distinguish between potential entry points for intervention.

To facilitate its use, we revised the study instrument to provide a simpler scoring system, along with a guide to its use (Additional file 4). It is worth noting that scores are added across items implying that each factor is weighted equally, which would favour the score towards organisational capabilities and likely reflect the relative (im)balance between factors in reality. We believe that the tool has the potential to provide quantitative benchmarks to measure MoH capacity to demand and use research over time and between countries. Further application of the instrument is needed to assess its usefulness as a tool for MoHs and those who support them to improve capacity to demand and use research, and its potential as an assessment applied over time.

### Limitations

There are several limitations that should be noted for this project. First, the framework assumes that the MoH and its individual constituents have at least some desire to use research evidence in their work. Second, the conceptual framework does not explicitly take into account the specific contextual factors that surround the MoH and their effect on this process, especially economic, social, legal/regulatory and political factors. Third, since the instrument is meant to have the potential to be applied across country settings, it was a challenge to create items that were sufficiently general, but also specific enough to discern variability in experiences. Fourth, the sample for this study has a few weaknesses. Four additional countries were to be included, but they did not complete the data collection. In two cases, issues with MoH approvals prevented data collection from starting, while in two others there were problems related with the study team’s ability to collect data (e.g. retirement of personnel). Whereas there is a limited number of countries involved in the study, the differences in size and MoH organisation do have some variability, which contributes to the broader applicability of the instrument. Additionally, there was considerable variability in the time between test and re-test for several respondents due to practical realities in getting busy officials to provide their time more than once. However, the tool is not meant to assess evidence at a specific point in time, and therefore we do not believe that the time lag in re-test would significantly affect responses. Finally, several country teams raised concerns about applying the instrument only in English if the country had other official language(s). In order to ensure that the instrument was applied consistently across settings, the research team had to insist on English-only application. Now that the instrument is finalised in one language, translations can be developed and validated further.

## Conclusion

This article describes the development and conceptualisation of the capacities necessary for MoHs to demand and use research evidence in their decision-making by (1) providing a more detailed view of the steps taken in evidence use and the skills and capabilities necessary at multiple levels to support it, and (2) validating a tool to assess the MoH’s capacity. The conceptual framework forms the basis for future assessment, understanding and improvement of the demand and use of research evidence that takes into account the complexities that decision-makers face, while the tool presents the opportunity for both internal discussions about how to build capacity in MoHs to make best use of available evidence as well as cross-country comparisons. For the national stakeholders, the final instrument and discussion questions will allow MoHs to assess themselves across capacity areas and identify future action points for improving capacity. It is hoped that this instrument will be regarded as a valuable tool for officials in MoHs to identify intervention points to strengthen their demand and use of research evidence in their decisions.

## Additional files

Additional file 1: Publications abstracted during literature review. (DOC 136 kb)

Additional file 2: Publications reviewed in detail. (DOCX 16 kb)

Additional file 3: Test-retest correlation results (*n* = 24). (DOCX 14 kb)

Additional file 4: Final tool and sampling rubric. (DOCX 25 kb)
