# Supplementary material for: Assessing the capacity of ministries of health to use research in decision-making: conceptual framework and tool
Source: Health Res Policy Syst. 2017 Aug 1;15:65. doi: 10.1186/s12961-017-0227-3 (PMC5539643; doi:10.1186/s12961-017-0227-3)
Supplement: Supplementary file 1 — Publications abstracted during literature review. (DOC 136 kb) [file 12961_2017_227_MOESM1_ESM.doc]

**Supplementary File. Publications Abstracted during Literature Review**

| **Documents Abstracted** |
| --- |
| Albert, M. A., A. Fretheim, et al. (2007). "Factors influencing the utilization of research findings by health policy-makers in a developing country: the selection of Mali's essential medicines." Health research policy and systems / BioMed Central 5: 2. |
| Alvaro, C., R. F. Lyons, et al. (2010). "Conservation of resources theory and research use in health systems." Implementation Science 5. |
| Andrus, J. K., B. Jauregui, et al. (2011). "Challenges to building capacity for evidence-based new vaccine policy in developing countries." Health Affairs 30(6): 1104-1112. |
| Andrus, J. K., C. M. Toscano, et al. (2007). "A model for enhancing evidence-based capacity to make informed policy decisions on the introduction of new vaccines in the Americas: PAHO's ProVac initiative." Public Health Reports 122(6): 811-816. |
| Ashford, L. S., R. R. Smith, et al. (2006). "Creating windows of opportunity for policy change: Incorporating evidence into decentralized planning in Kenya." Bulletin of the World Health Organization 84(8): 669-672. |
| Behague, D., C. Tawiah, et al. (2009). "Evidence-based policy-making: the implications of globally-applicable research for context-specific problem-solving in developing countries." Social science & medicine 69(10): 1539-1546. |
| Beier, J. C., J. Keating, et al. (2008). "Integrated vector management for malaria control." Malaria Journal 7(SUPPL. 1). |
| Bickford, J. J. and A. R. Kothari (2008). "Research and knowledge in Ontario Tobacco control networks." Canadian Journal of Public Health 99(4): 297-300. |
| Blume, S. and J. Tump (2010). "Evidence and policymaking: The introduction of MMR vaccine in the Netherlands." Social science & medicine 71(6): 1049-1055. |
| Boyko, J. A., J. N. Lavis, et al. (2011). "Reliability of a tool for measuring theory of planned behaviour constructs for use in evaluating research use in policymaking." Health research policy and systems / BioMed Central 9(1): 29. |
| Brousselle, A. and C. Lessard (2011). "Economic evaluation to inform health care decision-making: promise, pitfalls and a proposal for an alternative path." Social science & medicine 72(6): 832-839. |
| Burns, J. E., R. C. Mitrovich, et al. (2009). "Descriptive analysis of immunization policy decision making in the Americas." Revista Panamericana de Salud Publica/Pan American Journal of Public Health 26(5): 398-404. |
| Cameron, D., J. N. Lavis, et al. (2010). "Bridging the gaps among research, policy and practice in ten low- and middle-income countries: development and testing of a questionnaire for researchers." Health research policy and systems / BioMed Central 8(1): 4. |
| Chambers, D., P. M. Wilson, et al. (2011). "Maximizing the impact of systematic reviews in health care decision making: a systematic scoping review of knowledge-translation resources." The Milbank quarterly 89(1): 131-156. |
| Coffman, J. M., M. K. Hong, et al. (2009). "Translating medical effectiveness research into policy: lessons from the California Health Benefits Review Program." The Milbank quarterly 87(4): 863-902. |
| Colon-Ramos, U., A. C. Lindsay, et al. (2007). "Translating research into action: a case study on trans fatty acid research and nutrition policy in Costa Rica." Health Policy and Planning 22(6): 363-374. |
| Contandriopoulos, D., M. Lemire, et al. (2010). "Knowledge exchange processes in organizations and policy arenas: a narrative systematic review of the literature." The Milbank quarterly 88(4): 444-483. |
| Court, J. and S. Maxwell (2005). "Policy entrepreneurship for poverty reduction: Bridging research and policy in international development." Journal of International Development 17(6): 713-725. |
| Crites, G. E., M. C. McNamara, et al. (2009). "Evidence in the learning organization." Health Research Policy and Systems 7. |
| Daniels, K. and S. Lewin (2008). "Translating research into maternal health care policy: a qualitative case study of the use of evidence in policies for the treatment of eclampsia and pre-eclampsia in South Africa." Health research policy and systems / BioMed Central 6: 12. |
| de Goede, J., K. Putters, et al. (2010). "Knowledge in process? Exploring barriers between epidemiological research and local health policy development." Health research policy and systems / BioMed Central 8: 26. |
| de Leeuw, E. (2009). "Evidence for Healthy Cities: reflections on practice, method and theory." Health Promotion International 24 Suppl 1: i19-i36. |
| Dobbins, M., P. Rosenbaum, et al. (2007). "Information transfer: what do decision makers want and need from researchers?" Implementation Science 2. |
| Dobrow, M. J., V. Goel, et al. (2006). "The impact of context on evidence utilization: a framework for expert groups developing health policy recommendations." Social science & medicine 63(7): 1811-1824. |
| Dobrow, M. J., V. Goel, et al. (2004). "Evidence-based health policy: context and utilisation." Social science & medicine 58(1): 207-217. |
| Ettelt, S. and N. Mays (2011). "Health services research in Europe and its use for informing policy." Journal of Health Services Research and Policy 16(SUPPL. 2): 48-60. |
| European Commission (2005). Institutional Assessment and Capacity Development: Why, what and how? Tools and Methods Series: Reference Document No. 1. EuropeAid. Luxembourg, European Commission. |
| Flitcroft, K., J. Gillespie, et al. (2011). "Getting evidence into policy: The need for deliberative strategies?" Social Science and Medicine 72(7): 1039-1046. |
| French, B., L. H. Thomas, et al. (2009). "What can management theories offer evidence-based practice? A comparative analysis of measurement tools for organisational context." Implementation Science 4. |
| Fretheim, A., S. Munabi-Babigumira, et al. (2009). "SUPPORT tools for evidence-informed policymaking in health 6: Using research evidence to address how an option will be implemented." Health research policy and systems / BioMed Central 7 Suppl 1: S6. |
| Fretheim, A., A. D. Oxman, et al. (2009). "SUPPORT tools for evidence-informed policymaking in health 18: Planning monitoring and evaluation of policies." Health research policy and systems / BioMed Central 7 Suppl 1: S18. |
| Gholami, J., R. Majdzadeh, et al. (2011). "How should we assess knowledge translation in research organizations; designing a knowledge translation self-assessment tool for research institutes (SATORI)." Health research policy and systems / BioMed Central 9: 10. |
| Gilbert, K. L., S. C. Quinn, et al. (2011). "The urban context: A place to eliminate health disparities and build organizational capacity." Journal of Prevention and Intervention in the Community 39(1): 77-92. |
| Gilson, L. and D. McIntyre (2008). "The interface between research and policy: experience from South Africa." Social science & medicine 67(5): 748-759. |
| Ginsburg, L. R., S. Lewis, et al. (2007). "Revisiting interaction in knowledge translation." Implementation Science 2. |
| Gonzalez Block, M. A. and A. Mills (2003). "Assessing capacity for health policy and systems research in low and middle income countries*." Health research policy and systems / BioMed Central 1(1): 1. |
| Gordon, E. J. (2006). "The political contexts of evidence-based medicine: policymaking for daily hemodialysis." Social science & medicine 62(11): 2707-2719. |
| Green, A. E. and G. A. Aarons (2011). "A comparison of policy and direct practice stakeholder perceptions of factors affecting evidence-based practice implementation using concept mapping." Implementation Science 6. |
| Guindon, G. E., J. N. Lavis, et al. (2010). "Bridging the gaps among research, policy and practice in ten low- and middle-income countries: development and testing of questionnaire for health-care providers." Health research policy and systems / BioMed Central 8(1): 3. |
| Gyapong, J. O., R. A. Selby, et al. (2011). "Challenges in linking health research to policy: A commentary on developing a multi-stakeholder response to orphans and vulnerable children in Ghana." Health Research Policy and Systems 9(SUPPL. 1). |
| Hamel, N. and T. Schrecker (2011). "Unpacking capacity to utilize research: A tale of the Burkina Faso public health association." Social Science and Medicine 72(1): 31-38. |
| Hanney, S. R. and M. A. Gonzalez-Block (2011). "Yes, research can inform health policy; but can we bridge the 'Do-Knowing It's Been Done' gap?" Health research policy and systems / BioMed Central 9: 23. |
| Hanney, S. R., M. A. Gonzalez-Block, et al. (2003). "The utilisation of health research in policy-making: concepts, examples and methods of assessment." Health research policy and systems / BioMed Central 1(1): 2. |
| Hanusaik, N., J. L. O'Loughlin, et al. (2010). "Organizational capacity for chronic disease prevention: A survey of Canadian public health organizations." European Journal of Public Health 20(2): 195-201. |
| Harpham, T. and T. Tuan (2006). "From research evidence to policy: Mental health care in Viet Nam." Bulletin of the World Health Organization 84(8): 664-668. |
| Helfrich, C. D., Y. F. Li, et al. (2009). "Organizational readiness to change assessment (ORCA): Development of an instrument based on the Promoting Action on Research in Health Services (PARIHS) framework." Implementation Science 4. |
| Hornby, P. and H. S. R. Perera (2002). "A development framework for promoting evidence-based policy action: Drawing on experiences in Sri Lanka." International Journal of Health Planning and Management 17(2): 165-183. |
| Hutchinson, E., B. Droti, et al. (2011). "Translating evidence into policy in low-income countries: lessons from co-trimoxazole preventive therapy." Bulletin of the World Health Organization 89(4): 312-316. |
| Hyder, A. A., A. Corluka, et al. (2011). "National policy-makers speak out: are researchers giving them what they need?" Health Policy and Planning 26(1): 73-82. |
| Jansen, M. W., H. A. van Oers, et al. (2010). "Public health: disconnections between policy, practice and research." Health research policy and systems / BioMed Central 8: 37. |
| Jauregui, B., A. Sinha, et al. (2011). "Strengthening the technical capacity at country-level to make informed policy decisions on new vaccine introduction: Lessons learned by PAHO's ProVac Initiative." Vaccine 29(5): 1099-1106. |
| Jbilou, J., N. Amara, et al. (2007). "Research-based-decision-making in Canadian Health Organizations: A behavioural approach." Journal of Medical Systems 31(3): 185-196. |
| Jenkins, R., W. Gulbinat, et al. (2004). "The Mental Health Country Profile: Background, design and use of a systematic method of appraisal." International Review of Psychiatry 16(1-2): 31-47. |
| Jewell, C. and L. Bero (2007). "Public participation and claimsmaking: Evidence utilization and divergent policy frames in California's ergonomics rulemaking." Journal of Public Administration Research and Theory 17(4): 625-650. |
| Jewell, C. J. and L. A. Bero (2008). ""Developing good taste in evidence": facilitators of and hindrances to evidence-informed health policymaking in state government." The Milbank quarterly 86(2): 177-208. |
| Jonsson, K., G. Tomson, et al. (2007). "Health systems research in Lao PDR: capacity development for getting research into policy and practice." Health research policy and systems / BioMed Central 5: 11. |
| Kothari, A., N. Edwards, et al. (2009). "Is research working for you? validating a tool to examine the capacity of health organizations to use research." Implementation Science 4(1). |
| Landry, R., N. Amara, et al. (2006). "The knowledge-value chain: A conceptual framework for knowledge translation in health." Bulletin of the World Health Organization 84(8): 597-602. |
| Landry, R., M. Lamari, et al. (2003). "The extent and determinants of the utilization of university research in government agencies." Public Administration Review 63(2): 192-205. |
| Lavis, J. N., J. A. Boyko, et al. (2009). "SUPPORT Tools for evidence-informed health Policymaking (STP) 14: Organising and using policy dialogues to support evidence-informed policymaking." Health research policy and systems / BioMed Central 7 Suppl 1: S14. |
| Lavis, J. N., J. Lomas, et al. (2006). "Assessing country-level efforts to link research to action." Bulletin of the World Health Organization 84(8): 620-628. |
| Lavis, J. N., A. D. Oxman, et al. (2009). "SUPPORT Tools for evidence-informed health Policymaking (STP) 7: Finding systematic reviews." Health research policy and systems / BioMed Central 7 Suppl 1: S7. |
| Lavis, J. N., A. D. Oxman, et al. (2009). "SUPPORT Tools for evidence-informed health Policymaking (STP)." Health research policy and systems / BioMed Central 7 Suppl 1: I1. |
| Lavis, J. N., A. D. Oxman, et al. (2009). "SUPPORT Tools for evidence-informed health Policymaking (STP) 3: Setting priorities for supporting evidence-informed policymaking." Health research policy and systems / BioMed Central 7 Suppl 1: S3. |
| Lavis, J. N., A. D. Oxman, et al. (2008). "Evidence-informed health policy 1 - Synthesis of findings from a multi-method study of organizations that support the use of research evidence." Implementation Science 3(1). |
| Lavis, J. N., A. D. Oxman, et al. (2009). "SUPPORT Tools for evidence-informed health Policymaking (STP) 9: Assessing the applicability of the findings of a systematic review." Health research policy and systems / BioMed Central 7 Suppl 1: S9. |
| Lavis, J. N., E. J. Paulsen, et al. (2008). "Evidence-informed health policy 2-Survey of organizations that support the use of research evidence." Implementation Science 3. |
| Lavis, J. N., G. Permanand, et al. (2009). "SUPPORT Tools for evidence-informed health Policymaking (STP) 13: Preparing and using policy briefs to support evidence-informed policymaking." Health research policy and systems / BioMed Central 7 Suppl 1: S13. |
| Lavis, J. N., D. Robertson, et al. (2003). "How can research organizations more effectively transfer research knowledge to decision makers?" The Milbank quarterly 81(2): 221-248, 171-222. |
| Lavis, J. N., M. G. Wilson, et al. (2009). "SUPPORT Tools for evidence-informed health Policymaking (STP) 5: Using research evidence to frame options to address a problem." Health research policy and systems / BioMed Central 7 Suppl 1: S5. |
| Lavis, J. N., M. G. Wilson, et al. (2009). "SUPPORT Tools for evidence-informed health Policymaking (STP) 4: Using research evidence to clarify a problem." Health research policy and systems / BioMed Central 7 Suppl 1: S4. |
| Lewin, S., A. D. Oxman, et al. (2009). "SUPPORT Tools for evidence-informed health Policymaking (STP) 8: Deciding how much confidence to place in a systematic review." Health research policy and systems / BioMed Central 7 Suppl 1: S8. |
| Lewin, S., A. D. Oxman, et al. (2009). "SUPPORT tools for evidence-informed policymaking in health 11: Finding and using evidence about local conditions." Health research policy and systems / BioMed Central 7 Suppl 1: S11. |
| Loevinsohn, B. P. (1994). "Data utilization and analytical skills among mid-level health programme managers in a developing country." International Journal of Epidemiology 23(1): 194-200. |
| Lomas, J. and A. D. Brown (2009). "Research and advice giving: a functional view of evidence-informed policy advice in a Canadian Ministry of Health." The Milbank quarterly 87(4): 903-926. |
| Majdzadeh, R., S. Nedjat, et al. (2009). "Iran's approach to knowledge translation." Iranian Journal of Public Health 38(SUPPL. 1): 58-62. |
| Majdzadeh, R., B. Yazdizadeh, et al. (2011). "Strengthening evidence-based decision-making: is it possible without improving health system stewardship?" Health Policy and Planning. |
| Mason, R. (1976). Maximizing the use of research in population program and policy development in Pakistan, USAID. |
| McAneney, H., J. F. McCann, et al. (2010). "Translating evidence into practice: a shared priority in public health?" Social science & medicine 70(10): 1492-1500. |
| McCaughey, D. and N. S. Bruning (2010). "Rationality versus reality: the challenges of evidence-based decision making for health policy makers." Implementation Science 5. |
| MEASURE Evaluation (2006). Data Demand and Information Use in the Health Sector: Conceptual Framework. Chapel Hill, NC, Carolina Population Center at the University of North Carolina at Chapel Hill. |
| MEASURE Evaluation (2006). Data Demand and Information Use in the Health Sector: Strategies and Tools. Chapel Hill, NC, Carolina Population Center at the University of North Carolina at Chapel Hill. |
| Mitton, C., C. E. Adair, et al. (2007). "Knowledge transfer and exchange: review and synthesis of the literature." The Milbank quarterly 85(4): 729-768. |
| Mitton, C. and S. Patten (2004). "Evidence-based priority-setting: What do the decision-makers think?" Journal of Health Services Research and Policy 9(3): 146-152. |
| Munira, S. L. and S. A. Fritzen (2007). "What influences government adoption of vaccines in developing countries? A policy process analysis." Social science & medicine 65(8): 1751-1764. |
| Nath, S. (2007). Final Report: Getting Research into Policy and Practice (GRIPP), USAID, JSI Europe and Population Council. |
| Ouimet, M., R. Landry, et al. (2006). "What factors induce health care decision-makers to use clinical guidelines? Evidence from provincial health ministries, regional health authorities and hospitals in Canada." Social science & medicine 62(4): 964-976. |
| Oxman, A. D., A. Fretheim, et al. (2009). "SUPPORT Tools for evidence-informed health Policymaking (STP) 12: Finding and using research evidence about resource use and costs." Health research policy and systems / BioMed Central 7 Suppl 1: S12. |
| Oxman, A. D., A. Fretheim, et al. (2006). "Improving the use of research evidence in guideline development: introduction." Health research policy and systems / BioMed Central 4: 12. |
| Oxman, A. D., A. Fretheim, et al. (2006). "Improving the use of research evidence in guideline development: Introduction." Health Research Policy and Systems 4. |
| Oxman, A. D., J. N. Lavis, et al. (2009). "SUPPORT Tools for evidence-informed health Policymaking (STP) 16: Using research evidence in balancing the pros and cons of policies." Health research policy and systems / BioMed Central 7 Suppl 1: S16. |
| Oxman, A. D., J. N. Lavis, et al. (2009). "SUPPORT Tools for evidence-informed health Policymaking (STP) 17: Dealing with insufficient research evidence." Health research policy and systems / BioMed Central 7 Suppl 1: S17. |
| Oxman, A. D., J. N. Lavis, et al. (2009). "SUPPORT Tools for evidence-informed health Policymaking (STP) 1: What is evidence-informed policymaking?" Health research policy and systems / BioMed Central 7 Suppl 1: S1. |
| Oxman, A. D., J. N. Lavis, et al. (2009). "SUPPORT Tools for evidence-informed health Policymaking (STP) 10: Taking equity into consideration when assessing the findings of a systematic review." Health research policy and systems / BioMed Central 7 Suppl 1: S10. |
| Oxman, A. D., S. Lewin, et al. (2009). "SUPPORT Tools for evidence-informed health Policymaking (STP) 15: Engaging the public in evidence-informed policymaking." Health research policy and systems / BioMed Central 7 Suppl 1: S15. |
| Oxman, A. D., P. O. Vandvik, et al. (2009). "SUPPORT Tools for evidence-informed health Policymaking (STP) 2: Improving how your organisation supports the use of research evidence to inform policymaking." Health research policy and systems / BioMed Central 7 Suppl 1: S2. |
| Pappaioanou, M., M. Malison, et al. (2003). "Strengthening capacity in developing countries for evidence-based public health: the data for decision-making project." Social science & medicine 57(10): 1925-1937. |
| Perrier, L., K. Mrklas, et al. (2011). "Interventions encouraging the use of systematic reviews by health policymakers and managers: A systematic review." Implementation Science 6. |
| Potter, C. and R. Brough (2004). "Systemic capacity building: a hierarchy of needs." Health Policy and Planning 19(5): 336-345. |
| Schünemann, H. J., A. Fretheim, et al. (2006). "Improving the use of research evidence in guideline development: 13. Applicability, transferability and adaptation." Health Research Policy and Systems 4. |
| Seedat, M. and A. Nascimento (2003). "The use of public health research in stimulating violence and injury prevention practices and policies: Reflections from South Africa." Journal of Prevention and Intervention in the Community 25(1): 31-47. |
| Senouci, K., J. Blau, et al. (2010). "The Supporting Independent Immunization and Vaccine Advisory Committees (SIVAC) Initiative: A country-driven, multi-partner program to support evidence-based decision making." Vaccine 28(SUPPL. 1): A26-A30. |
| Solo, J., S. Harbison, et al. (2008). CRTU Research Utilization Assessment Report - Research with a purpose. The Global Health Technical Assistance Project. Washington, DC, USAID. |
| Stetler, C. B., J. A. Ritchie, et al. (2009). "Institutionalizing evidence-based practice: an organizational case study using a model of strategic change." Implementation Science 4. |
| Stewart, J. and T. Raiwaqavuka (2009). "Working with a community controlled health organization to develop an evidence base for their practice models." Australasian Psychiatry 17(SUPPL. 1): S79-S82. |
| Theobald, S. and B. Nhlema-Simwaka (2008). "The research, policy and practice interface: reflections on using applied social research to promote equity in health in Malawi." Social science & medicine 67(5): 760-770. |
| Thomson, H. (2008). "HIA forecast: Cloudy with sunny spells later?" European Journal of Public Health 18(5): 436-438. |
| Tomson, G., C. Paphassarang, et al. (2005). "Decision-makers and the usefulness of research evidence in policy implementation--a case study from Lao PDR." Social science & medicine 61(6): 1291-1299. |
| van Kammen, J., D. de Savigny, et al. (2006). "Using knowledge brokering to promote evidence-based policy-making: The need for support structures." Bulletin of the World Health Organization 84(8): 608-612. |
| Vondal, P. J., L. Cooley, et al. (1998). How Can Research Influence Policy Change? Evidence from Africa. Implementing Policy Change Project. USAID, USAID. |
| Wang, W., L. Saldana, et al. (2010). "Factors that influenced county system leaders to implement an evidence-based program: a baseline survey within a randomized controlled trial." Implementation Science 5. |
| Wilson, M. G., J. N. Lavis, et al. (2010). "Community-based knowledge transfer and exchange: Helping community-based organizations link research to action." Implementation Science 5. |
| Wilson, M. G., S. B. Rourke, et al. (2011). "Community capacity to acquire, assess, adapt, and apply research evidence: a survey of Ontario's HIV/AIDS sector." Implementation Science 6. |
| Wittroc, B. (1982). "Social Knowledge, Public Policy and Social Betterment: A Review of Current Research on Knowledge Utilization in Policy-Making." European Journal of Political Research 10(1): 83-89. |
| Woelk, G., K. Daniels, et al. (2009). "Translating research into policy: lessons learned from eclampsia treatment and malaria control in three southern African countries." Health research policy and systems / BioMed Central 7: 31. |
| Young, J. (2005). "Research, policy and practice: Why developing countries are different." Journal of International Development 17(6): 727-734. |
