# Supplementary material for: Assessing the capacity of ministries of health to use research in decision-making: conceptual framework and tool
Source: Health Res Policy Syst. 2017 Aug 1;15:65. doi: 10.1186/s12961-017-0227-3 (PMC5539643; doi:10.1186/s12961-017-0227-3)
Supplement: Supplementary file 2 — Publications reviewed in detail. (DOCX 16 kb) [file 12961_2017_227_MOESM2_ESM.docx]

**Supplementary File. Publications Reviewed in Detail**

| **Publication** | **Relevance to Project** | **Tool / Framework / High Ranking** |
| --- | --- | --- |
| **Boyko, J. A., J. N. Lavis, et al. (2011). "Reliability of a tool for measuring theory of planned behaviour constructs for use in evaluating research use in policymaking." Health research policy and systems / BioMed Central 9(1): 29.** | Tool measuring intention to use research evidence based on Theory of Planned Behavior | T |
| **Cameron, D., J. N. Lavis, et al. (2010). "Bridging the gaps among research, policy and practice in ten low- and middle-income countries: development and testing of a questionnaire for researchers." Health research policy and systems / BioMed Central 8(1): 4.** | Development and testing questionnaire focused on researchers' engagement in bridging activities related to high-priority health topics in LMICs | T |
| **Colon-Ramos, U., A. C. Lindsay, et al. (2007). "Translating research into action: a case study on trans fatty acid research and nutrition policy in Costa Rica." Health Policy and Planning 22(6): 363-374.** | Framework assessing factors process of evidence translation in policymaking in Costa Rica | F |
| **Court, J. and S. Maxwell (2005). "Policy entrepreneurship for poverty reduction: Bridging research and policy in international development." Journal of International Development 17(6): 713-725.** | Rapid Framework linking evidence, actors, context and influences | F |
| **Crites, G. E., M. C. McNamara, et al. (2009). "Evidence in the learning organization." Health Research Policy and Systems 7.** | Frameworks for organizational learning through evidence-based decision-making | F |
| **de Goede, J., K. Putters, et al. (2010). "Knowledge in process? Exploring barriers between epidemiological research and local health policy development." Health research policy and systems / BioMed Central 8: 26.** | Analtytical framework for research utilzation for Dutch policymaking | F |
| **Dobrow, M. J., V. Goel, et al. (2004). "Evidence-based health policy: context and utilisation." Social science & medicine 58(1): 207-217.** | Proposed framework for evidence-based decision-making focused on context | F |
| **Dobrow, M. J., V. Goel, et al. (2006). "The impact of context on evidence utilization: a framework for expert groups developing health policy recommendations." Social science & medicine 63(7): 1811-1824.** | Refined framework for expert policy groups developing health policy recommendations | F |
| **European Commission (2005). Institutional Assessment and Capacity Development: Why, what and how? Tools and Methods Series: Reference Document No. 1. EuropeAid. Luxembourg, European Commission.** | Capacity assessment at organizational level | HR |
| **French, B., L. H. Thomas, et al. (2009). "What can management theories offer evidence-based practice? A comparative analysis of measurement tools for organisational context." Implementation Science 4.** | Tools for capacity assessment at organizational and individual levels | T |
| **Gholami, J., R. Majdzadeh, et al. (2011). "How should we assess knowledge translation in research organizations; designing a knowledge translation self-assessment tool for research institutes (SATORI)." Health research policy and systems / BioMed Central 9: 10.** | Tool operationalizing knowledge translation model | T |
| **Gilson, L. and D. McIntyre (2008). "The interface between research and policy: experience from South Africa." Social science & medicine 67(5): 748-759.** | Example of research evidence use in South Africa | HR |
| **Gonzalez Block, M. A. and A. Mills (2003). "Assessing capacity for health policy and systems research in low and middle income countries*." Health research policy and systems / BioMed Central 1(1): 1.** | Organization and systems capacities | HR |
| **Hanney, S. R., M. A. Gonzalez-Block, et al. (2003). "The utilisation of health research in policy-making: concepts, examples and methods of assessment." Health research policy and systems / BioMed Central 1(1): 2.** | Conceptualization of research utilization | HR |
| **Hyder, A. A., A. Corluka, et al. (2011). "National policy-makers speak out: are researchers giving them what they need?" Health Policy and Planning 26(1): 73-82.** | Individual, organization and systems capacities | HR |
| **Kothari, A., N. Edwards, et al. (2009). "Is research working for you? Validating a tool to examine the capacity of health organizations to use research." Implementation Science 4(1).** | Tool to discuss and examine the capacity of health service organizations to use evidence | T |
| **Landry, R., M. Lamari, et al. (2003). "The extent and determinants of the utilization of university research in government agencies." Public Administration Review 63(2): 192-205.** | Individual capacity, incl. steps for research utilization | HR |
| **Majdzadeh, R., B. Yazdizadeh, et al. (2011). "Strengthening evidence-based decision-making: is it possible without improving health system stewardship?" Health Policy and Planning.** | Individual, organization and systems capacities | HR |
| **McCaughey, D. and N. S. Bruning (2010). "Rationality versus reality: the challenges of evidence-based decision making for health policy makers." Implementation Science 5.** | Individual capacity | HR |
| **MEASURE Evaluation (2006). Data Demand and Information Use in the Health Sector: Conceptual Framework. Chapel Hill, NC, Carolina Population Center at the University of North Carolina at Chapel Hill.** | Framework to improve information use in health sector in developing countries | F |
| **MEASURE Evaluation (2006). Data Demand and Information Use in the Health Sector: Strategies and Tools. Chapel Hill, NC, Carolina Population Center at the University of North Carolina at Chapel Hill.** | Discussion guide for assessing constraints affecting demand and supply of data | T |
| **Mitton, C. and S. Patten (2004). "Evidence-based priority-setting: What do the decision-makers think?" Journal of Health Services Research and Policy 9(3): 146-152.** | Individual and organization capacities | HR |
| **Mitton, C., C. E. Adair, et al. (2007). "Knowledge transfer and exchange: review and synthesis of the literature." The Milbank quarterly 85(4): 729-768.** | Individual and organization capacities | HR |
| **Ouimet, M., R. Landry, et al. (2006). "What factors induce health care decision-makers to use clinical guidelines? Evidence from provincial health ministries, regional health authorities and hospitals in Canada." Social science & medicine 62(4): 964-976.** | Individual and organization capacities | HR |
| **Oxman, A. D., P. O. Vandvik, et al. (2009). "SUPPORT Tools for evidence-informed health Policymaking (STP) 2: Improving how your organisation supports the use of research evidence to inform policymaking." Health research policy and systems / BioMed Central 7 Suppl 1: S2.** | Tool focused on organizational support for using evidence | T |
| **Potter, C. and R. Brough (2004). "Systemic capacity building: a hierarchy of needs." Health Policy and Planning 19(5): 336-345.** | Focused on capacity building from systems perspective | HR |
| **Tomson, G., C. Paphassarang, et al. (2005). "Decision-makers and the usefulness of research evidence in policy implementation--a case study from Lao PDR." Social science & medicine 61(6): 1291-1299.** | Organization and systems capacities | HR |
| **Wilson, M. G., S. B. Rourke, et al. (2011). "Community capacity to acquire, assess, adapt, and apply research evidence: a survey of Ontario's HIV/AIDS sector." Implementation Science 6.** | Organizational capacities based on 4 As (acquire, assess, adapt, apply) | HR |
| **Young, J. (2005). "Research, policy and practice: Why developing countries are different." Journal of International Development 17(6): 727-734.** | Systems capacity with focus on LMICs | HR |
