# Supplementary material for: Assessing the capacity of ministries of health to use research in decision-making: conceptual framework and tool
Source: Health Res Policy Syst. 2017 Aug 1;15:65. doi: 10.1186/s12961-017-0227-3 (PMC5539643; doi:10.1186/s12961-017-0227-3)
Supplement: Supplementary file 3 — Test-retest correlation results (n = 24). (DOCX 14 kb) [file 12961_2017_227_MOESM3_ESM.docx]

**Supplementary File. Test-retest correlation results (n=24)**

| Original Item Number | Test-Retest Correlation |
| --- | --- |
| 1 | 1.00 |
| 2 | 1.00 |
| 3 | 0.88 |
| 4 | 0.76 |
| 5 | 0.56* |
| 6 | 0.74 |
| 7 | 1.00** |
| 8 | 0.84 |
| 9 | 0.85 |
| 10 | 0.60* |
| 11 | 1.00 |
| 12 | 1.00 |
| 13 | 0.92 |
| 14a | 0.60*** |
| 14b | 0.90 |
| 14c | 1.00 |
| 15a | 0.80 |
| 15b | 1.00 |
| 15c | 0.95 |
| 16a | 0.56* |
| 16b | 1.00 |
| 16c | 0.77 |
| 17a | 0.65*** |
| 17b | 0.60* |
| 17c | 0.59*** |
| 18a | 1.00 |
| 18b | 0.71*** |
| 19a | 1.00 |
| 19b | 0.65*** |
| 20a | 1.00 |
| 20b | 0.61*** |
| 21a | 0.80 |
| 21b | 0.73*** |
| 22a | 1.00 |
| 22b | 0.84 |
| 23a | 1.00 |
| 23b | 0.84 |
| 24 | 0.69*** |
| 25 | 0.82 |
| 26 | 1.00 |
| 27 | 0.89 |
| 28 | 1.00** |
| 29 | 1.00** |
| 30 | 1.00 |
| 31 | 0.78** |
| 32 | 0.87 |
| 33 | 0.91 |
| 34 | 0.70*** |
| 35 | 0.91 |
| 36 | 0.62*** |
| 37 | 0.83 |
| 38 | 0.83 |
| 39 | 0.91 |
| 40 | 1.00 |
| 41 | 1.00 |
| 42 | 1.00 |
| 43 | 1.00 |
| 45a | 1.00 |
| 45b | 0.84 |
| 45c | 0.92 |
| 45d | 0.51* |
| 45e | 1.00 |
| 45f | 1.00 |
| 45g | 0.95 |
| 46a | 0.93 |
| 46b | 1.00 |
| 46c | 0.93 |
| 46d | 0.88 |
| 47 | 1.00 |
| 48 | 1.00 |
| 49 | 0.74*** |
| 50 | Could not be computed**** |
| 51 | 1.00 |
| 52 | 0.87 |
| 53 | Could not be computed**** |
| 54 | Could not be computed**** |
| * 2-sided p-value >0.10  ** 2-sided p-value >0.05  *** Correlation <0.80  **** Did not vary in this sub-sample, could not be computed | |
