# Supplementary material for: Assessing the capacity of ministries of health to use research in decision-making: conceptual framework and tool
Source: Health Res Policy Syst. 2017 Aug 1;15:65. doi: 10.1186/s12961-017-0227-3 (PMC5539643; doi:10.1186/s12961-017-0227-3)
Supplement: Supplementary file 4 — Final tool and sampling rubric. (DOCX 25 kb) [file 12961_2017_227_MOESM4_ESM.docx]

Supplementary File: Final Tool and Sampling Rubric

**Assessing Capacity of Ministries of Health to Use Research Evidence to Improve Decision-making**

**Introduction**

This tool has been designed to assess the capacity of Ministries of Health to demand and use research evidence to improve decision-making. This tool is intended to help identify gaps in capacity to use research evidence and opportunities for improvement.

There are two broad categories of capacity that are being assessed here: capacity at the organizational level, and capacity at the individual level. **Please answer the questions to the best of your knowledge and ability.** At the end of the tool there are a series of open questions to foster further discussion.

Thank you for taking the time to complete this tool.

**Demographic Questions**

1. Gender: Female Male
2. What is the main unit (division, department, or program) where you work in the MoH?
3. How long have you worked in the Ministry of Health?

______ months ______ years

**Section 1.**

The following questions are about the processes and practices that reflect Ministry of Health’s commitment to using research evidence in its decisions and for influencing others outside the Ministry, including allocation of staff for key steps.

*For each of these statements, please indicate your level of agreement.*

|  |  | **LOW HIGH** | | | |
| --- | --- | --- | --- | --- | --- |
|  | Using research evidence is a priority in the MoH. | 0 | 1 | 2 | 3 |
|  | Leadership in the MoH supports evidence-informed decisions. | 0 | 1 | 2 | 3 |
|  | Decision-makers in the MoH give consideration to any recommendations based on research evidence. | 0 | 1 | 2 | 3 |
|  | There is a transparent process for how research evidence is used in decisions at the MoH. | 0 | 1 | 2 | 3 |
|  | The MoH conducts activities to promote the use of research evidence. | 0 | 1 | 2 | 3 |
|  | The MoH has a process to check regularly whether I use research evidence in my work. | 0 | 1 | 2 | 3 |
|  | The MoH gets involved with researchers as partners in decision-making. | 0 | 1 | 2 | 3 |
|  | The MoH has a good process to advocate its priorities based on research evidence to the public, such as to promote behavior change. | 0 | 1 | 2 | 3 |
|  | The MoH has a good process to advocate its priorities based on research evidence to health workers, such as to promote changes in clinical practice. | 0 | 1 | 2 | 3 |
|  | The MoH has a good process to advocate its priorities based on research evidence to other ministries, such as to justify the costs of health interventions. | 0 | 1 | 2 | 3 |
|  | The MoH has a good process to advocate its priorities based on research evidence to professional organizations, such as to promote new roles for different health workers. | 0 | 1 | 2 | 3 |
|  | The current policy environment is supportive of the MoH using research evidence for its decisions. | 0 | 1 | 2 | 3 |
|  | The current government is supportive of the MoH using research evidence for its decisions. | 0 | 1 | 2 | 3 |
|  | Stakeholders outside the MoH actively engage the MoH to contribute research evidence to inform decisions. | 0 | 1 | 2 | 3 |
|  | MOH staff search for and retrieve research evidence for the MOH. | 0 | 1 | 2 | 3 |
|  | MOH staff interpret research evidence for the MOH. | 0 | 1 | 2 | 3 |
|  | MOH staff synthesize all the relevant research evidence, information and analyses for a specific issue. | 0 | 1 | 2 | 3 |
|  | MOH staff compare what the MoH does to what the research evidence says. | 0 | 1 | 2 | 3 |
|  | MOH staff link research evidence to key issues facing decision-makers. | 0 | 1 | 2 | 3 |
|  | MOH staff provide recommendations based on research evidence to decision-makers. | 0 | 1 | 2 | 3 |

**Section 2.**

The following questions are the resources necessary for Ministry of Health staff to assess research evidence and provide recommendations.

*For each of these statements, please indicate your level of agreement.*

|  |  | **LOW HIGH** | | | |
| --- | --- | --- | --- | --- | --- |
|  | Our staff has enough time to evaluate research evidence. | 1 | 2 | 3 | 4 |
|  | Our staff has enough resources to evaluate research evidence. | 1 | 2 | 3 | 4 |
|  | Our staff has enough time to compare what the Ministry of Health does to what the research evidence says. | 1 | 2 | 3 | 4 |
|  | Our staff has enough resources to compare what the Ministry of Health does to what the research evidence says. | 1 | 2 | 3 | 4 |
|  | Our staff has enough time to link research evidence to key issues facing decision-makers. | 1 | 2 | 3 | 4 |
|  | Our staff has enough resources to link research evidence to key issues facing decision-makers. | 1 | 2 | 3 | 4 |
|  | Our staff has enough time to provide recommendations based on research evidence to decision-makers. | 1 | 2 | 3 | 4 |
|  | Our staff has enough resources to provide recommendations based on research evidence to decision-makers. | 1 | 2 | 3 | 4 |
|  | Our unit has regular access to a computer for acquiring and analyzing research evidence. | 1 | 2 | 3 | 4 |
|  | Our unit has regular access to the Internet at work for accessing research evidence online. | 1 | 2 | 3 | 4 |

**Discussion Questions**

1. How is research evidence used to inform decisions? How could research evidence be used better to inform decisions?
2. What activities to promote research use are conducted by the MOH? How useful have they been?
3. Which MOH units most use research evidence currently? Which MOH units should use research evidence more? Why?
4. How does the MOH engage with outside stakeholders regarding research evidence and its use?
5. How is the MOH involved in setting priorities for research that can inform its decisions? How should they set research priorities more effectively?
6. Any other comments?

Sampling rubric

The following sampling rubric should be used to identify respondents in the Ministry of Health to complete this tool. This is a generic rubric that should be applied to the local Ministry organogram as appropriate.

| **MOH Unit** | **Type of respondents*** | **Number per unit** |
| --- | --- | --- |
| Ministerial level | Minister, Vice/Deputy Minister, Administrative head (e.g. Principal Secretary) and Head of Health Service (e.g. Director General), or their equivalents | 3-5 persons per MOH, depending on organization structure |
| Policy and Planning Unit (or equivalent) | Head and Deputy Head | 2 respondents |
| Research Unit (if one exists) | Head and Deputy Head | 2 respondents |
| Monitoring and Evaluation Unit | Head and Deputy Head | 2 respondents |
| Public Health Programs | Head and Deputy Head | 2 respondents |
| Specific health programs, if multiple programs (e.g. maternal/child health, nutrition, HIV)** | Head of program (random selection of 5 respondents from heads of such programs) | 5 respondents |
| Hospital Services (or health services) | Head and Deputy Head | 2 respondents |
| International Relations | Head of unit | 1 respondent |
| Regional/District units | Head of unit (random selection of 10 respondents from heads of such units) | 10 respondents |
| *For those units that have two deputy heads, ask both to participate. If there are more than 2 deputy heads, randomly sample two of them.  **In the specific health programs, identify at least four or five eligible divisions. | | |
